# Elevated serum levels of macrophage-derived chemokine and thymus and activation-regulated chemokine in autistic children

**DOI:** 10.1186/1742-2094-10-72

**Published:** 2013-06-19

**Authors:** Laila Yousef AL-Ayadhi, Gehan Ahmed Mostafa

**Affiliations:** 1 Autism Research and Treatment Center, AL-Amodi Autism Research Chair, Department of Physiology, Faculty of Medicine, King Saud University, Riyadh, Saudi Arabia; 2 Department of Pediatrics, Faculty of Medicine, Ain Shams University, 9 Ahmed El-Samman Street off Makram Ebaid, Nasr City, Cairo 11511, Egypt

**Keywords:** Autism, Autoimmunity, Childhood Autism Rating Scale, CCR4 ligands, Macrophage-derived chemokine, Thymus and activation-regulated chemokine

## Abstract

**Background:**

In some autistic children, there is an imbalance of T helper (Th)1/Th2 lymphocytes toward Th2, which may be responsible for the induction of the production of autoantibodies in these children. Th2 lymphocytes express CCR4 receptors. CCR4 ligands include macrophage-derived chemokine (MDC) and thymus and activation-regulated chemokine (TARC). They direct trafficking and recruitment of Th2 cells. We are the first to measure serum levels of CCR4 ligands in relation to the degree of the severity of autism.

**Methods:**

Serum concentrations of MDC and TARC were measured, by quantitative sandwich enzyme immunoassay technique, in 56 autistic children and 32 healthy matched children.

**Results:**

Autistic children had significantly higher serum levels of MDC and TARC than healthy controls (*P* <0.001 and *P* <0.001, respectively). Children with severe autism had significantly higher serum levels of MDC and TARC than patients with mild to moderate autism (*P* <0.001 and *P* = 0.01, respectively). In addition, there were significant positive correlations between CARS and serum levels of both MDC (*P* <0.001) and TARC (*P* <0.001) in children with autism. There were significant positive correlations between serum levels of MDC and TARC in autistic children (*P* <0.001).

**Conclusions:**

Serum levels of CCR4 ligands were elevated in autistic children and they were significantly correlated to the degree of the severity of autism. However, further research is warranted to determine the pathogenic role of CCR4 ligands in autism and to shed light on the therapeutic role of CCR4-ligand antagonism in autistic children.

## Background

Recruitment of inflammatory cells plays the key pathogenic role in all inflammatory and autoimmune diseases. Inflammation is characterized by the local tissue expression of chemokines, a large group of chemotactic cytokines, which play an important pathogenic role in inflammatory diseases by enhancement of leukocyte recruitment and activation at inflammatory sites [1-4].

CCR4 is a receptor that binds two 8 kDa CC chemokines, macrophage-derived chemokine (MDC/CCL22) and thymus and activation-regulated chemokine (TARC/CCL17) [5]. MDC is synthesized by cells of macrophage lineage, while TARC is expressed in the thymus. The gene encoding both chemokines is clustered on chromosome 16q13 [6-8]. CCR4 ligands, MDC and TARC, act on their CCR4 receptors to enhance the recruitment and activation of T helper (Th) 2 cells with a subsequent production of type 2 cytokines that include interleukin-4 (IL-4), IL-5, IL-9 and IL-13 [9,10]. The Th2-associated chemokines, MDC and TARC, were the highest at birth and then decreased with age [11]. CCR4 ligands have an important pathogenic role in inflammatory conditions such as allergy and some autoimmune diseases [12-18]. Chemokines and their receptors have been implicated as functional mediators of immunopathology of autoimmune neuroinflammatory diseases [19,20].

The presence of autoantibodies to neural tissues/antigens in autism [21-28] and the increase in the frequency of autoimmune disorders among autistic families [29-33] suggest that autoimmunity may play an important role in the pathogenesis of autism [21].

This study aimed to measure serum levels of CCR4 ligands in relation to the degree of the severity of autism in a group of autistic children.

## Methods

### Study population

This cross-sectional study was conducted on 56 autistic children. They were recruited from the Autism Research and Treatment Center, Faculty of Medicine, King Saud University, Riyadh, Saudi Arabia. Patients fulfilled the criteria of the diagnosis of autism according to the 4th edition of the Diagnostic and Statistical Manual of Mental Disorders [34]. The autistic group comprised 46 males and 10 females. Their ages ranged between 4 and 12 years (mean ± SD = 7.54 ± 1.96 years). Patients who had associated neurological diseases (such as cerebral palsy and tuberous sclerosis), metabolic disorders (for example phenylketonuria), allergic manifestations or concomitant infection were excluded from the study.

The control group comprised 32 age- and sex-matched apparently healthy children. They included 26 males and 6 females. They were the healthy older siblings of the healthy infants who attend the Well Baby Clinic, King Khalid University Hospital, Faculty of Medicine, King Saud University, Riyadh, Saudi Arabia for the routine following up of their growth parameters. The control children were not related to the children with autism, and demonstrated no clinical findings suggestive of infections, allergic manifestations and immunological or neuropsychiatric disorders. Their ages ranged between 5 and 11 years (mean ± SD = 7.19 ± 1.69 years). The local Ethical Committee of the Faculty of Medicine, King Saud University, Riyadh, Saudi Arabia, approved this study. In addition, an informed written consent of participation in the study was signed by the parents or the legal guardians of the studied subjects.

### Study measurements

#### Clinical evaluation of autistic patients

This was based on clinical history taking from caregivers, clinical examination and neuropsychiatric assessment. In addition, the degree of the disease severity was assessed by using the Childhood Autism Rating Scale (CARS) [35] which rates the child on a scale from one to four in each of fifteen areas (relating to people; emotional response; imitation; body use; object use; listening response; fear or nervousness; verbal communication; nonverbal communication; activity level; level and consistency of intellectual response; adaptation to change; visual response; taste, smell and touch response and general impressions). According to the scale, children who have scored 30 to 36 have mild to moderate autism (n = 20), while those with scores ranging between 37 and 60 points have a severe degree of autism (n = 36).

#### Serum assessment of MDC and TARC levels

The assay employed the quantitative sandwich enzyme immunoassay technique using the Quantikin Human MDC/CCL22 and TARC-CCL17 immunoassay kits (both kits were supplied from R&D Systems Inc., Minneapolis, MN, USA). A monoclonal antibody specific for MDC or TARC had been precoated onto a microplate. Standards and samples were pipetted into the wells and any MDC or TARC present was bound by the immobilized antibody. After washing away any unbound antibody-enzyme reagent, a substrate solution was added to the wells and color developed in proportion to the amount of MDC or TARC bound in the initial step. The color development was stopped and the intensity of the color was measured [7,16]. To increase accuracy, all samples were analyzed twice in two independent experiments to assess the interassay variations and to ensure reproducibility of the observed results. There were no discordant data between the results (*P* >0.05). No significant cross-reactivity or interference was observed.

### Statistical analysis

The results were analyzed by using the commercially available software package (Statview, Abacus Concepts, Inc., Berkley, CA, USA). The data were nonparametric, thus they were presented as median and interquartile range (IQR), which are between the 25th and 75th percentiles. The Mann–Whitney test was used for comparison between these data. A chi-square test was used for comparison between qualitative variables of the studied groups. Spearman’s rho correlation coefficient ‘r’ was used to determine the relationship between different variables. For all tests, a probability (*P*) of less than 0.05 was considered significant.

## Results

**Figure 1:**
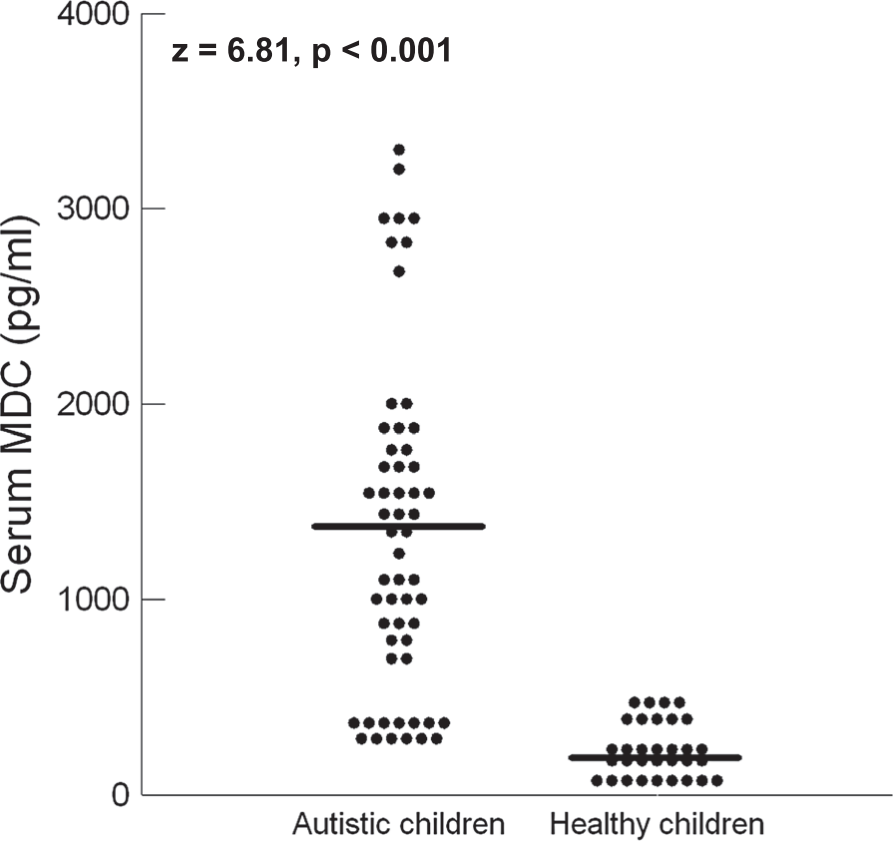
**Serum levels of MDC in autistic patients and healthy controls.** MDC, macrophage-derived chemokine. Horizontal bars indicate the median values.

**Figure 2:**
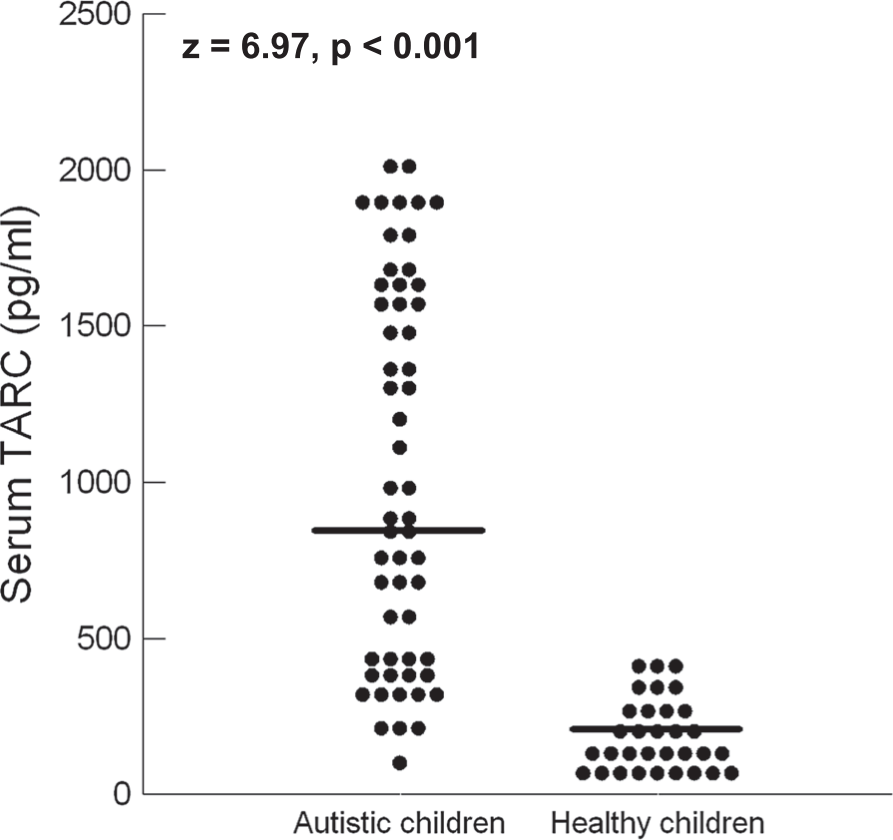
**Serum levels of TARC in autistic patients and healthy controls.** TARC, thymus and activation-regulated chemokine. Horizontal bars indicate the median values. TARC, thymus and activation-regulated chemokine.

Autistic children had significantly higher serum levels of MDC (median (IQR) = 1289.5 (1079) pg/ml) than healthy controls (median (IQR) = 190 (295) pg/ml), *P* <0.001 (Figure 1). Similarly, autistic children had significantly higher serum levels of TARC (median (IQR) = 880.5 (1206) pg/ml) than healthy children (median (IQR) = 137.25 (173) pg/ml), *P* <0.001, (Figure 2).

Children with severe autism had significantly higher serum levels of MDC and TARC than patients with mild to moderate autism (P < 0.001 and P = 0.01, respectively), Table 1. In addition, there were significant positive correlations between CARS and serum levels of both MDC (r = 0.93, P < 0.001) and TARC (r = 0.87, P < 0.001) in children with autism.

**Table 1:** Serum levels of MDC and TARC in relation to the severity of autism

**Serum CCR4 ligands**	**Patients with mild to moderate autism (n = 20)**	**Patients with severe autism (n = 36)**	**Z (P-value)**
	**Median (IQR)**	**Median (IQR)**	
Serum MDC (pg/ml)	767.5 (649)	634 (451)	4.8 (< 0.001)
Serum TARC (pg/ml)	1623 (651)	1469 (1217)	3.2 (0.01)

*MDC* Macrophage-derived chemokine, *TARC* thymus and activation-regulated chemokine.

**Figure 3:**
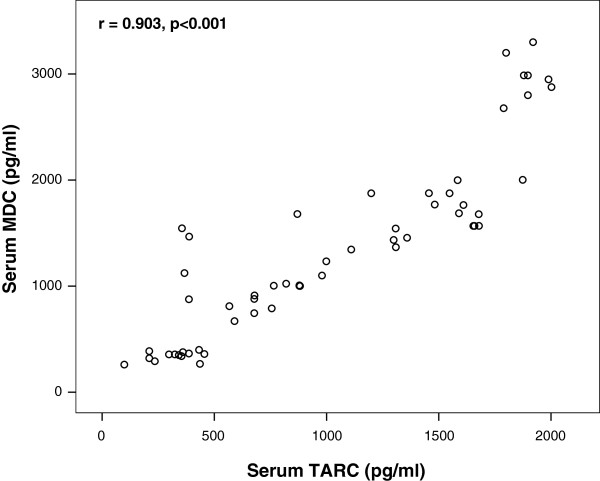
**Positive correlations between serum levels of MDC and TARC in autistic patients.** MDC, macrophage-derived chemokine, TARC, thymus and activation- regulated chemokine.

There were significant positive correlations between serum levels of MDC and TARC in autistic children, P < 0.001 (Figure 3).

Serum levels of both MDC and TARC had no significant correlations with the age of the children with autism (*P* = 0.87 and *P* = 0.47), respectively. In addition, there was no significant difference between male and female autistic children in serum levels of both MDC (*P* >0.05) and TARC (*P* >0.05).

All study subjects had normal weight (body mass index (BMI) was between the 5th and less than the 85th percentiles based on age and sex). There was no significant difference between the BMI of healthy children and autistic children (*P* >0.05). In addition, there were no significant correlations between the BMI and serum levels of both MDC (*P* >0.05) and TARC (*P* >0.05) in children with autism.

## Discussion

CCR4 ligands direct trafficking and recruitment of Th2 cells [9,10] and they were reported to play an important role in some autoimmune diseases [15-18]. In the present study, autistic children had significantly higher serum levels of MDC and TARC than healthy controls (*P* <0.001 and *P* <0.001, respectively). A previous study reported increased MDC and TARC in the anterior cingulate gyrus of autistic patients [36]. Proinflammatory chemokines, such as TARC and monocyte chemotactic protein-1 (MCP-1) along with proinflammatory cytokines were reported to be elevated in the brains of some individuals with autism.

The transport or synthesis of cytokines in the brain may contribute to neuroinflammation and possible neurotransmitter imbalances in autism [21]. Abnormal immune responses, as assessed by multiplex analysis of cytokines and chemokines, may serve as one of the biological trait markers for autism [37]. Active neuroinflammatory process in the cerebral cortex, white matter, and notably in cerebellum of autistic patients was reported. Immunocytochemical studies showed marked activation of microglia and astroglia, and cytokine profiling indicated that macrophage chemoattractant protein-1 and tumor growth factor-beta1, derived from neuroglia, were the most prevalent cytokines in brain tissues. Cerebrospinal fluid (CSF) showed a unique proinflammatory profile of cytokines, including a marked increase in MCP-1 [36].

Elevated levels of chemokines have been detected in the brain and CSF of individuals with autism. Few studies have examined chemokine levels in the plasma of children with autism, but these studies did not correlate the plasma levels of chemokines with the disease severity. Elevated plasma MCP-1, RANTES and eotaxin in some autistic children and their association with more impaired behaviors may have etiological significance. Chemokines and their receptors might provide unique targets for future therapies in autism [38]. Another study reported decreased plasma levels of chemokines involved in hematopoiesis and the Th cell immune system in the children with autism compared with unrelated siblings without autism. The investigators of this study recommended further studies to confirm immunological disturbances influencing hematopoiesis and antibody production in the children with autism [39]. An increased risk for infantile autism with elevated MCP-1 in amniotic fluid was recently reported. Elevated levels of MCP-1 may play an indirect role in the pathophysiology of autism [40].

This study is the first to investigate serum levels of CCR4 ligands in relation to the degree of the severity of autism, which was assessed by using CARS. In the present work, children with severe autism had significantly higher serum levels of MDC than patients with mild to moderate autism (*P* <0.001 and *P* = 0.01, respectively). There were significant positive correlations between the values of CARS and serum levels of both MDC (*P* <0.001) and TARC (*P* <0.001) in autistic children. This may indicate that the extent of the elevation of CCR4 ligands was closely linked to the degree of the severity of autism. However, it is not easy to determine whether this increase is a mere consequence of autism or has a pathogenic role in the disease. Further research is warranted to determine the pathogenic role of CCR4 ligands and their relation to serum levels of brain-specific autoantibodies in autistic children.

CCR4 ligands have many cellular sources including monocyte-derived dendritic cells, keratinocytes and bronchial epithelial cells [41-43]. MDC is also produced by natural killer cells and macrophages [44]. Early signals elicited during innate immune response, such as Th2-type cytokines and bacterial products, induce the rapid secretion of CCR4 [45,46]. The current study revealed significant positive correlations between serum MDC and serum TARC levels in autistic children (*P* <0.001). This could be explained by the fact that MDC is produced concomitantly with TARC during immune inflammation. Both chemokines are ligands to the same receptor, which is characterized on Th2 cells [9] resulting in the activation and long-term recruitment of Th2 cells [47] and subsequently in the pathomechanism of some autoimmune diseases, including autism [15-17].

Both Th2 chemoattractants, MDC and TARC, were reported to be elevated and play a role in some autoimmune diseases such as systemic lupus erythematosus [15] and systemic sclerosis [16]. In addition, MDC levels were reported to be elevated in the CSF of female patients with multiple sclerosis (MS). MDC was suggested to play a role in the development of MS possibly by influencing the intracerebral recruitment of Th2 cells which express CCR4 receptors [17]. Furthermore, MDC plays a role in experimental autoimmune encephalomyelitis (EAE), the animal model of MS. MDC/CCL22 gene is a part of a chemokine cluster, which includes also TARC/CCL17. The frequency of the C/T and C/A single nucleotide polymorphisms (SNPs) in the promoter and coding sequence of CCL22 as well as the C/T SNP in the promoter of CCL17 were determined in 370 patients with MS compared with 380 controls. A trend toward a decreased allelic frequency of the A allele of the CCL22 C/A SNP as well as of the T allele of the CCL17 C/T SNP was found in patients compared with controls [19]. Similar genetic studies of MDC/CCL22 gene are recommended in children with autism.

The immunomodulatory effects of CCR 4 ligands may act not only through modulation of Th cell function, but also through decreasing the induction of CD4 + CD25high regulatory T cells (T-regs) and the promotion of the differentiation of Th17 cells [48]. Th17 cells have been implicated in the pathogenesis of autoimmune diseases, which is supported by recent clinical trials using anti-IL-17 in the treatment of these diseases. Th17 cells are characterized by a strong IL-17-producing capacity. IL-17 family cytokines control the inflammatory responses by triggering the secretion of proinflammatory cytokines and chemokines [49,50]. In addition, T-regs play an important role in the establishment of the immunological self-tolerance and thereby, prevent autoimmunity as T-regs suppress Th17 cells [51]. One study reported deficiency of T-regs in 73.3% of autistic children [32]. Another studies reported significantly higher plasma concentrations of IL-17 [37,52] and some of Th2 cytokines, such as IL-5 and IL-13, in autistic children compared to healthy matched controls [37,38].

Previous studies reported the possible pathogenic role of Th2 cells, Th17 cells and deficiency of T-regs in the induction of autoimmunity in a subgroup of patients with autism [32,35,36,50]. The results of this study may indicate the possible contributing role of CCR4 ligands in the induction of autoimmunity in autism, possibly as a result of long-term recruitment of Th2 cells, suppression of the induction of T-regs and enhancement of the differentiation of Th17 cells. However, this is an initial report that cannot prove the pathogenic role of CCR4 ligands in autism, but rather raises additional questions. So, these data should be treated with caution until further investigations are performed. Studies should be conducted to investigate the relationship between serum levels of CCR4 ligands and both T-regs and Th17 cells in children with autism.

Decreasing chemokine receptor binding may be an important potential therapeutic target in allergy and immunology [4]. This can be performed by using small molecule chemokine receptors antagonists or by using blocking antibodies [53]. Thus, studies concerning the effect of CCR4-ligand antagonism on amelioration of autistic manifestations in children are mandatory.

## Conclusions

Serum levels of CCR4 ligands were elevated in autistic children and they were significantly correlated to the degree of the severity of autism. However, further research is warranted to determine the pathogenic role of CCR4 ligands in autism and to shed light on the therapeutic role of CCR4-ligand antagonism in autistic children.

## Abbreviations

BMI: Body mass index; CARS: Childhood Autism Rating Scale; EAE: Experimental autoimmune encephalomyelitis; IL: Interleukin; IQR: Interquartile range; MDC: Macrophage-derived chemokine; MCP-1: Monocyte chemotactic protein-1; SNPs: Single Nucleotide Polymorphisms; TARC: Thymus and activation- regulated chemokine; Th: T-helper; MS: Multiple sclerosis; Tregs: CD4 + CD25 high regulatory T-cells.

## Competing interests

The authors declare that they have no competing interests.

## Authors’ contributions

Both authors designed, performed and wrote the research. In addition, both authors read and approved the final manuscript.
